# Increased Baseline Pupil Size Linked to Uncertainty Avoidance in Decision Making

**DOI:** 10.1111/ejn.70394

**Published:** 2026-01-28

**Authors:** Ehsan Kakaei, Anne Schlecht, Tobias U. Hauser

**Affiliations:** ^1^ Department of Psychiatry and Psychotherapy, Faculty of Medicine University Tübingen Tübingen Germany; ^2^ Functional Imaging Laboratory (FIL), Department of Imaging Neuroscience University College London London UK; ^3^ Max Planck UCL Centre for Computational Psychiatry and Ageing Research University College London London UK; ^4^ Partner Site Tübingen German Center for Mental Health (DZPG) Tübingen Germany

**Keywords:** cognition, noradrenaline, pupil dilation

## Abstract

Uncertainty is a key contributor to decision making, and humans show inconsistent attitudes towards it. Although excessive uncertainty‐avoidance or uncertainty‐seeking are hallmark symptoms of several mental conditions, the neural mechanism underlying uncertainty seeking and avoidance remains unclear. Here, we probed whether changes in pupil‐linked arousal are indicative of uncertainty avoidance in humans. Investigating baseline pupil size to capture endogenous fluctuations across two experiments (*N*_1_ = 24, *N*_2_ = 21), we found that pretrial pupillary responses (as early as 700 ms prior to the onset of a trial) were closely related to uncertainty attitudes during multiarmed bandit tasks. Although increased baseline pupil size signalled avoidance in uncertainty‐related decisions, it did not foreshadow value processing per se. The specificity of our results suggests that uncertainty processing is dynamic and depends on (potentially noradrenergic) endogenous pupil fluctuations.

## Graphical abstract

Pupil dialation as early as ~1 s prior to an uncertain choice signals uncertainty avoidant behaviour, choosing certainty over rewards.

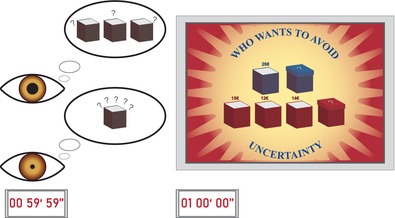

**Abbreviations**
NA: noradrenaline
SEM: standard error mean

## 1 Introduction

Making a good decision in uncertain environments is challenging and—at times—even scary. Should you bet on safety and repeat your previous actions, which proved to be rewarding enough, or should you risk it and try a new option, hoping to gain more? Interestingly, humans differ greatly in such situations (Hau et al. 2008; Kelly and Sharot 2021), and distinct fields highlight opposite behavioural strategies. Although humans often avoid the unknown (uncertainty avoidance; Tversky and Kahneman 1981), under certain conditions, humans appear to embrace these uncertainties (uncertainty seeking; Dubois et al. 2021; Dubois and Hauser 2022; Roberts 1991; Tversky and Kahneman 1981). Although there is a considerable literature linking individual differences to these different uncertainty‐related strategies (Dubois et al. 2021; Dubois and Hauser 2022), little is known about the consistency of these strategies within a single person.

Previous behavioural, computational and neural studies have distinguished distinct uncertainty‐related decision‐making strategies and have linked them to different underlying neural processes. Uncertainty avoidance is often seen in decisions with risky or ambiguous choices and has been linked to multiple clinical conditions including anxiety, depression and obsessive‐compulsive disorder (for a review, see Attaallah et al. 2025). An inflated estimation of uncertainty could partially explain uncertainty‐avoidant behaviour in these conditions (Attaallah et al. 2025; Grupe and Nitschke 2013). In contrast, ambiguity‐related uncertainty seeking is often seen in the context of exploration‐exploitation trade‐offs, where the potential of gaining information is believed to guide behaviour towards more informative choices (Auer 2003; Gershman 2018; Schwartenbeck et al. 2019; Speekenbrink and Konstantinidis 2015) or renders choices more stochastic (Dubois et al. 2021; Dubois and Hauser 2022; Fan et al. 2023; Wilson et al. 2014). Recent studies have linked this uncertainty‐embracing behaviours to impulsive traits (Dubois et al. 2022; Dubois and Hauser 2022), addictive behaviours (Attaallah et al. 2025; Verdejo‐Garcia et al. 2018) and delusional disorders (Attaallah et al. 2025; Bentall and Swarbrick 2003; Moritz et al. 2007; Moutoussis et al. 2011; Roberts 1991).

Although the neural mechanisms underlying this uncertainty processing are not entirely clear, several theoretical and empirical insights have suggested that the noradrenaline (NA) system plays a crucial role. NA is believed to signal different forms of uncertainty (Aston‐Jones and Cohen 2005; Dayan and Yu 2006; Doya 2008; Yu and Dayan 2005). Pharmacological studies have shown that noradrenergic drugs change how humans process uncertainty (Dubois et al. 2021; Hauser et al. 2018). Pupil‐linked arousal—a hypothesised marker for noradrenaline activity (Bang et al. 2023; Joshi et al. 2016; Joshi and Gold 2020; Murphy et al. 2014)—has also been suggested to modulate uncertainty processing (Fan et al. 2023; Jepma and Nieuwenhuis 2011).

Critically, brain and neurotransmitter activity is known to constantly fluctuate, even in the absence of tasks or during rest. Such fluctuations can alter both low‐ and high‐level processes and partly explain the variable and inconsistent behaviour in humans (Boly et al. 2007; Chew et al. 2019; Fox et al. 2006; Sadaghiani et al. 2015). Here, we hypothesised that uncertainty‐based decisions may be under noradrenergic control and fluctuations in this neurotransmitter system change the impact of uncertainty on decision making, even within short periods of seconds and minutes. To this end, we assessed the impact of endogenous pupil size fluctuations as an indirect proxy for noradrenaline, and we show that these fluctuations can partly explain within‐subject variability in ambiguity‐related uncertainty avoidant behaviour.

## 2 Materials and Methods

### 2.1 Participants

We analysed data from 45 healthy subjects (24 subjects in Experiment 1 and 21 subjects in Experiment 2) aged between 19 and 41 (25 years ± 4.5, mean ± SD). Subjects were reimbursed for their attendance €8–10 per hour plus maximum of €5 bonus depending on their performance. Subjects were recruited, provided informed consent, were debriefed and reimbursed for their attendance in accordance with the approved local ethics (No. 268/2023BO2).

### 2.2 Setup and Apparatus

Subjects took part in the experiments in a dark room with dimmed lights. This setup was kept constant throughout the experiment to avoid external influences on (baseline) pupil size. Subjects placed their chin and fixed their head on a chinrest while performing tasks. A monitor (1920 × 1080 resolution, 52.7 × 29.6 cm dimension, gamma 2.5 and maximum luminance of 350 cd/m^2^) was placed 106 cm away from the subjects (corresponding to maximum visual angle of 28°). We obtained monocular pupil diameter using SR‐research Eyelink 1000 at a sampling rate of 1000 Hz and calibrated the gaze position.

### 2.3 Paradigm

#### 2.3.1 Experiment 1

We employed a modified version of a previously established multiarmed bandit task (Dubois et al. 2021; Dubois and Hauser 2022; Wilson et al. 2014) during which subjects observed three stationary bandits (i.e., fixed average outcome within a trial) with unbalanced certainty ℂ (i.e., number of pre‐drawn samples) AND expected values v associated with each bandit, so we could probe the baseline pupil's contribution to different decision strategies (e.g., value‐based vs. uncertainty‐avoidant decision making). Importantly, uncertainty is characterised here as ambiguity, rather than explicit risk (e.g., probability of a reward).

For each trial, we generated the values assigned to each bandit $i\in \left\{1,2,3\right\}$ with the expected reward ($\mu_{i}$) using a Gaussian distribution $N\left(\mu_{i},\sigma \right)$. The distance between expected reward values was constant ($\mu_{1}>\mu_{2}>\mu_{3};\mu_{1}-\mu_{2}=8$ and $\mu_{2}-\mu_{3}=16$), and the variance was fixed for all three bandits (σ=14). All the values v were truncated to the range $v\in \left\{10,99\right\}$. At the beginning of each trial, we provided subjects with ℂ=5 predrawn samples such that the high‐value bandit (i=1) had ${\mathbb{C}}_{1}=1\;\text{or}\;3$ sample(s), the midvalue bandit (i=2) had ${\mathbb{C}}_{1}=3\;\text{or}\;1$ sample(s) and the low‐value bandit had ${\mathbb{C}}_{3}=1$ sample.

#### 2.3.2 Task Procedure

**FIGURE 1:**
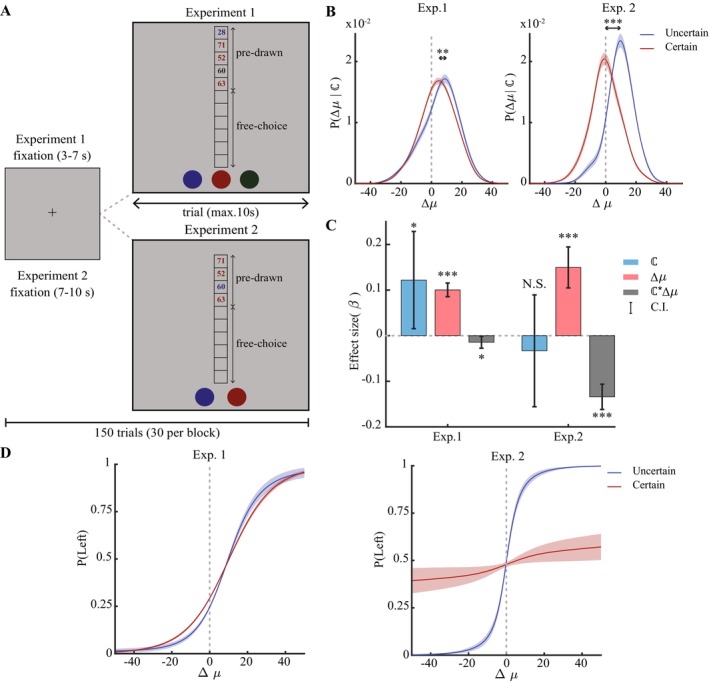
Experimental paradigm and the behavioural results. (A) Each experiment consisted of 150 trials across five blocks. Prior to each trial, subjects saw a fixation cross (3–7 s in Experiment 1 and 7–10 s in Experiment 2) followed by predrawn samples (five in Experiment 1 and four in Experiment 2), which were presented in random order and in different colours associated with each bandit (Experiment 1: blue, red and green; Experiment 2: blue and red). Subjects had maximum of 10 s to make six self‐paced free choices. (B) We calculated the conditional $P\left(\Delta \mu |\mathbb{C}\right)$ probability of draws over difference in the means $\Delta \mu =\mu_{\text{chosen}}-\mu_{\text{notchosen}}$ for each bandit with a given number of predrawn samples, under both Experiment 1 (left) and Experiment 2 (right). When the relative value of the choice was lower than the value of the alternative bandit (Δμ<0), subjects chose the *certain* bandits (i.e., bandits with more predrawn samples) more often, whereas the *uncertain* bandits (i.e., bandits with less predrawn samples) were chosen mostly when they carried higher value than the alternative (Δμ>0). This ‘shift’ to higher values was significant in both experiments. (C) To estimate contribution of the number and value of the predrawn samples to choice, we modelled probability of choosing the bandit on the left, as a function of the certainty (ℂ) and the difference in the means Δμ using a mixed‐effect generalised linear model. The estimated $\beta_{\Delta \mu }$ and the interaction parameter $\beta_{\mathbb{C}*\Delta \mu }$ were significant in both experiments, meaning that the magnitude difference matters for decision making but it governs the decision making less for *certain* options. We additionally found that *uncertain* options were generally avoided in experiment 1 ($\beta_{\mathbb{C}}$). (D) The fitted mixed‐effect model predicted the probability of choosing the left bandit over the difference in the means Δμ (psychometric curve), for each subject independently. The average choice probability of the certain bandit (higher number of predrawn samples) is less sensitive to the difference in the observed mean than the uncertain bandit. This can be seen as a flatter psychometric curve in both Experiment 1 (left) and Experiment 2 (right). *p < 0.05, **p < 0.01 and ***p < 0.001.

Each block started with a black cross (+) presented in the middle of the screen on a grey background during which subjects were instructed to fixate on the cross, followed by the predrawn samples presented in the colour associated with that bandit's colour (Figure 1A). Based on these predrawn samples, subjects were instructed to make six consecutive choices (horizon of 6) in 10 s to maximise their total reward for each trial. The outcomes of each of the six choices were displayed immediately after that choice in that bandit's colour. When subjects did not complete all six choices in 10 s, the trial timed out and a red cross (X) appeared on the screen in 0.6 ± 0.1% of the trials (mean ± SEM). Subjects completed 150 such trials across five blocks (30 trials per block). The colours (red, green and blue) and positions of the bandits (right, middle or left) were randomly assigned in each trial. Intertrial interval (ITI) was jittered between 3 and 7 s.

#### 2.3.3 Experiment 2

The paradigm in Experiment 2 was similar to the paradigm in Experiment 1, except that we employed a two‐armed bandit task with unbalanced certainty (${\mathbb{C}}_{1}=3,{\mathbb{C}}_{2}=1$, total predrawn samples of ℂ=4) OR expected values associated with each bandit ($\mu_{1}>\mu_{2};\mu_{1}-\mu_{2}=8;{\mathbb{C}}_{1}={\mathbb{C}}_{2}=2$). By doing so, we could dissociate pupillary responses prior to choice under value‐based or uncertainty‐avoidant strategies, separately.

#### 2.3.4 Task Procedure

The procedure of Experiment 2 was similar to the procedure in Experiment 1. Subjects completed 150 trials in five blocks (30 trials per block). The colours (red or blue) and positions of the bandits (right or left) were randomly assigned in each trial. The ITI jitters were increased to 7–10 s to further minimise any temporal overlap between pupillary responses of consecutive trials.

### 2.4 Behavioural Analysis

A common challenge for exploration tasks is a collinearity between uncertainty and value. We thus capitalised on a task and analysis design that delineates these two (for discussion, see Wilson et al. 2014). Critically, this means that we only analysed the first draws subjects took, in line with previous studies (Dubois et al. 2021; Dubois and Hauser 2022; Wilson et al. 2014). This design choice was also reflected in task design where subsequent choices followed in quick succession, making them indistinguishable for pupillometry‐related analyses.

We first grouped subjects' choices based on number of predrawn samples into *certain* and *uncertain* choices. A choice is an *uncertain* one if it has ℂ=1 predrawn sample and is a *certain* one if ℂ=3 predrawn samples. For each choice type ($\mathbb{C}\in \left\{1,3\right\}$), we calculated the probability distribution $P\left(\Delta \mu |\mathbb{C}\right)$ of the first draw over the difference between the average predrawn values of the chosen and not chosen options ($\Delta \mu =\mu_{\text{chosen}}-\mu_{\text{notchosen}}$) (Figure 1B). We tested the significance of the shift between the two probability distributions by first calculating the most likely Δμ (peak of the conditional probability) for the *certain* and *uncertain* choices, then calculating *t* static of the shift ($t\left(\Delta \mu_{\text{certain}}<\Delta \mu_{\text{uncertain}}\right)$) using a paired *t* test. We evaluated the contribution of number and values of the predrawn samples to choice, by modelling the probability of choosing the bandit on the left, as a function of the number of predrawn samples (ℂ) and the difference in the means Δμ using a mixed‐effect generalised linear model ($\text{logit}\left(P_{\text{left}}\right)\sim \beta_{0}+\beta_{\mathbb{C}}\;\mathbb{C}+\beta_{\Delta \mu }\;\Delta \mu +\beta_{\mathbb{C}\cdot \Delta \mu }\;\left(\mathbb{C}*\Delta \mu \right)+\left(\mathbb{C}*\Delta \mu |\text{subject}\right)+\epsilon$). The mixed‐effect model fits a psychometric curve in form of a sigmoid function for each subject separately. We averaged the fitted functions to generate an average psychometric curve for certain and uncertain choices (Figure 1D).

### 2.5 Pupil Preprocessing

Raw pupil data were parsed and segmented into epochs with a duration of 13 s (3 s pretrial to 10 s posttrial) using field‐trip toolbox (Oostenveld et al. 2011). Corrupted data points such as blinks, missing pupil data and irregular pupil sizes (e.g., pupil size when subjects squint their eyes) were corrected by a linear interpolation connecting the timepoints prior to and after each event, in accordance with our previous studies (Allen et al. 2016; Hauser et al. 2019). Within each experimental block, the interpolated data were detrended and whitened (i.e., zero meaned and unit variance).

### 2.6 Pupillometry Analysis

**FIGURE 2:**
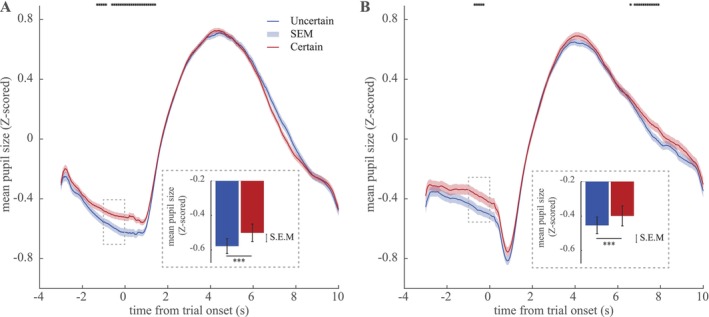
Baseline pupil size drives uncertainty avoidance. Pupil response from 3 s prior to trial onset till 10 s after the onset were *z* scored and grouped by the first choices' prior information. The average pupil response (± S.E.M) are shown for *certain* (red) and *uncertain* (blue) bandits during Experiment 1 (A) and Experiment 2 (B). The baseline pupillary responses (time window [−1, 0] s; see inset bar plots) prior to choosing *certain* bandits was significantly higher than prior to choosing *uncertain* bandits (for illustration purposes, uncorrected timepoint‐specific comparisons are shown in black *p < 0.05). In both Experiments 1 and 2, subjects were more likely to select the *certain* bandit when if followed an increased baseline pupil size. The error bars represent the standard error of mean (SEM). ***p < 0.001.

To examine the relation of baseline pupil size (*Y*) and the certainty level (ℂ), we grouped the pupillary responses of all trials based on subjects' *certain* or *uncertain* choices and modelled the pupil size at each given time point (*t*) as a function of certainty level using a linear mixed‐effect model ($Y_{\left\{t\right\}}\sim \beta_{0}+\beta_{\mathbb{C}}\mathbb{C}+\left(1|\text{subject}\right)+\epsilon_{t}$). Additionally, we modelled the pupillary responses at each given time point within a priori time window $t\in \left\{-1,0\right\}$ s prior to onset of the trial (i.e., when subjects saw all the options right after the fixation period) as a function of choice type. Because pupil responses are inherently autocorrelated (e.g., Zénon 2017), we tried to factor out this autocorrelation by considering time *t* as a random effect, and we assigned to the pupillary responses at each time point a different intercept. This corresponds to the linear mixed‐effect model ($Y_{\left\{t\right\}}\sim \beta_{0}+\beta_{\mathbb{C}}\mathbb{C}+\left(1|\text{subject}\right)+\left(1|t\right)+\epsilon_{t}$). Results of these analyses are reported in Figure 2.

**FIGURE 3:**
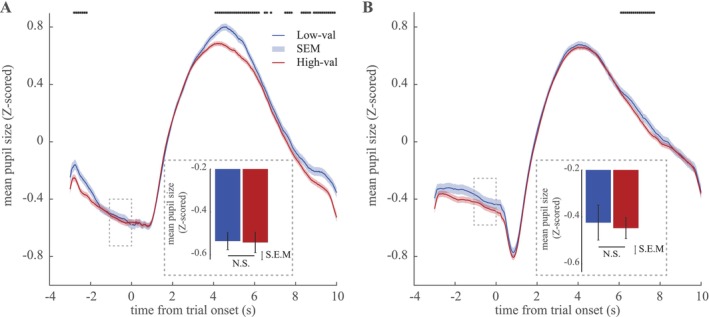
No baseline pupil effects related to value‐based decision making. Baseline pupil response (mean ± 1 SEM) did not differ between high‐value (red) and low‐value (blue) bandits during Experiment 1 (A) and Experiment 2 (B). The baseline pupillary responses (time window [−1, 0] s; see inset bar plots) prior to choosing bandits with higher predrawn values over lower values were not significantly different. Uncorrected differences (*p* < 0.05) are illustrated by black *.

We used similar approaches to model correlation of the baseline pupil size with the bandits' values, by grouping trials based on observed mean value of the first draw values (high value: Δμ *>* 0 vs. low value: Δμ < 0). We modelled the pupil size as a function of value group (*V*) both in a time‐point specific ($Y_{\left\{t\right\}}\sim \beta_{0}+\beta_{V}V+\left(1|\text{subject}\right)+\epsilon_{t}$) and in a priori window ($Y_{\left\{t\right\}}\sim \beta_{0}+\beta_{V}V+\left(1|\text{subject}\right)+\left(1|t\right)+\epsilon_{t}$). Results of these analyses are reported in Figure 3.

## 3 Results

The goal of these studies was to assess the relationship between baseline pupil fluctuations and uncertainty processing. To this end, we experimentally controlled for the certainty level (number of predrawn samples) and the expected values (reward magnitude) of each bandit prior to subjects' first free choice. By focusing only on subjects' first draw, we assured independence of certainty and value variables and avoided complications arising from analysis of further free‐choices (see Dubois et al. 2021; Dubois and Hauser 2022; Wilson et al. 2014).

### 3.1 Experiment 1

#### 3.1.1 Uncertainty Avoidance Guides Decision Making

We assessed the strategy subjects took on their first draw, by calculating the conditional probability $P\left(\Delta \mu |\mathbb{C}\right)$ of draws over the difference in the bandit's values, given the number of predrawn samples. Specifically, for each choice type ($\mathbb{C}\in \left\{1,3\right\}$), we calculated the probability distribution $P\left(\Delta \mu |\mathbb{C}\right)$ of the first draw over the difference between the average predrawn values of the chosen and not chosen options ($\Delta \mu =\mu_{\text{chosen}}-\mu_{\text{notchosen}}$). We found that it was more likely that subjects chose the *certain* bandit (i.e., bandit with more predrawn samples) even at the expense of choosing the lower value bandit ($\int_{-\infty }^{0}P\left(\Delta \mu |\text{certain}\right)\mathrm{d\mu}>\int_{-\infty }^{0}P\left(\Delta \mu |\text{uncertain}\right)\mathrm{d\mu}$; Figure 1B). Subjects tended to select the bandit with higher uncertainty (i.e., bandit with lower predrawn samples) mostly when this bandit was of higher value (Δμ>0). Indeed, the peak of *certain* bandit distribution was significantly (*t*[46] = 3.2, *p* < 0.01) lower that of the *uncertain* bandit. This suggests that subjects add more value for certainty or penalise uncertainty, given that they chose the bandits with lower uncertainty even when it had a lower expected value.

To disentangle effect of value and certainty on subjects' choices, we modelled the contribution of certainty (ℂ), difference in the observed prior means (Δμ) and their interaction (ℂ*Δμ) to probability of choosing the bandit on the left, using a linear mixed effect model. We found that subjects' choices were positively influenced by the difference in the observed mean ($\beta_{\Delta \mu }>0,t\left(2999\right)=13.2,p\sim 10^{-38},\mathrm{CI}=\left[0.09,0.11\right]$) and certainty level ($\beta_{\mathbb{C}}>0,t=2.2,p=0.025,\mathrm{CI}=\left[0.02,0.23\right]$), indicating that bandits with higher certainty and/or with higher value were more likely to be drawn (Figure 1C). We also found out that the interaction term between certainty and value was negative ($\beta_{\mathbb{C}*\Delta \mu }<0,t=-2.2,p=0.03,\mathrm{CI}=\left[-0.03,-0.001\right]$). This means that participants were willing to forego expected points if the bandit was more certain, in line with an uncertainty aversion. This was also visible when plotting the choice curves (Figure 1D), where choices of the certain bandit were less deterministic, indicating that participants were willing to forego rewards for certainty.

#### 3.1.2 Baseline Pupil Size Drives Uncertainty Avoidance

To assess how baseline pupil size is related to the uncertainty‐based decision strategy, we grouped the pupillary responses by the first choices' certainty level (*certain* vs. *uncertain*, Figure 2A). At each given time point, we quantified how pupil size can be explained by the level of certainty of the subsequent choice (Figure 2A) using a mixed‐effect generalised linear model (see Section 2). We observed a marked difference in baseline pupil size from −1.3 s onwards, lasting past trial onset until 1.4 s. This means that pupil size was larger prior (i.e., in the fixation period before trial onset) to *certain* than to *uncertain* choices. To test this more formally, we assessed baseline pupil size in our a priori baseline time window −1 to 0 s and found a highly significant difference in pupil size preceding *certain* vs. *uncertain* choices ($\beta_{\mathbb{C}}>0,t=6.9,p\sim 10^{-12},\mathrm{CI}=\left[0.03,0.05\right]$) (Figure 2A, inset bar plots).

To assess whether this baseline effect was specific to uncertainty, or whether it also carried information about the impact of expected value (i.e., magnitude of prior draws), we split the pupillary responses by their subsequent choice (choosing option with prior high value: Δμ *>* 0 vs. low value: Δμ *<* 0) and assessed the contribution of value to pupil size using a similar mixed‐effect model (Figure 3A). We did not observe any baseline pupil difference between high and low value choices, neither in our a priori baseline window ([−1, 0] s; Figure 3A, inset bar plots) nor any extended other temporal cluster ($\beta_{v}\approx 0,t=-0.6,p=0.56,\mathrm{CI}=\left[-0.01,0.01\right]$).

### 3.2 Experiment 2

Next, to further dissociate the contribution of the certainty from the effect of the bandit's value to the decision strategy, we conducted a second experiment by modifying the number of bandits and balancing either the certainty or the value of the bandits. This allowed us to replicate and refine our results.

#### 3.2.1 Uncertainty Modulates Use of Value

As in Experiment 1, we observed a significant (p<0.001,t=13.7) shift in probability distribution, meaning that subjects were choosing *certain* bandits with lower values than *uncertain* bandits (Figure 1B). This, again, is indicative of an uncertainty avoidance bias in this task. We also evaluated the contribution of value, certainty and their interaction to choice probability using a linear mixed model; once again, we observed a significant positive effect by the difference in the observed mean value ($\beta_{\Delta \mu }>0,t=6.5,p\sim 10^{-10},\mathrm{CI}=\left[0.1,0.2\right]$) and a negative interaction between value and certainty ($\begin{array}{c} \beta_{\mathbb{C}*\Delta \mu }<0,t=-9.5,p\sim 10^{-21}, \end{array}\begin{array}{c} \mathrm{CI}=\left[-0.16,-0.1\right] \end{array}$) (Figure 1C). In contrast to what we observed in the first experiment, the main effect of the certainty was not significant $\left(\beta_{\mathbb{C}}\approx 0,p=0.6,t=-0.5\right)$. This could be due to presence of trials in which both bandits had equal level of certainty, reducing number of trials with unbalanced certainty and possibly reducing sensitivity of the choice to certainty levels. Similar to experiment one, the model‐derive choice propensities (Figure 1D) also showed a reduced sensitivity to reward differences for the certain choices.

#### 3.2.2 Baseline Pupil Again Drives Uncertainty Avoidance

We repeated our pupillometry analysis similar to that of Experiment 1, by modelling the pupil size at each time point as a function of levels of certainty (Figure 2B), or as a function of difference in the observed mean value (Figure 3B). We again found a significant effect in our a priori baseline window ([−1, 0] s) ($\beta_{\mathbb{C}}>0,t=3.7,p\sim 10^{-4},\mathrm{CI}=\left[0.01,0.04\right]$), replicating the effect of increased pupil size prior to *certain* than *uncertain* choices. This was particularly prominent from −0.7 to −0.3 s.

When investigating the value effect, we again did not observe any baseline pupil effect between high‐ and low‐value bandits ($\beta_{v}\approx 0,t=-1.4,p=0.17,\mathrm{CI}=\left[-0.02,0.003\right]$), in line with the null findings in Experiment 1 (Figure 3B).

These results confirm the observation of the first experiment regarding the change in the baseline pupil size prior to a certainty‐based decision, with higher pupil size driving subjects' tendency to avoid uncertainty, but not prior to a value‐based decision.

## 4 Discussion

In this study, we examined how baseline pupil‐linked arousal, a proxy of endogenous noradrenaline fluctuations, is linked with value and uncertainty processing during decision making. We show that subjects integrate both value and certainty to guide their choice. Importantly, our results demonstrate that this varies within single subjects and that baseline pupil‐linked arousal foreshadows the impact of uncertainty (but not value) on decision making: Enlarged baseline pupil size preceded uncertainty‐avoiding choices, whereas lower baseline pupil signalled uncertainty‐seeking behaviour. This suggests that pupil‐size fluctuations, and the likely underlying NA activity, might be governing the balance between uncertainty‐seeking and uncertainty‐avoiding behaviour.

Previous works on the exact role of NA in decision‐making are not conclusive because some findings indicate an increased uncertainty‐seeking (Dubois et al. 2021; Hauser et al. 2018; Jepma and Nieuwenhuis 2011), whereas others show increased uncertainty‐avoidant (Cremer et al. 2023; Warren et al. 2017) behaviour associated with increased NA. Here, we found an uncertainty‐avoidant behaviour associated with momentarily increased NA, assuming that pupil size is an indirect measure of NA activity (Joshi et al. 2016; Joshi and Gold 2020). Our findings expand on this literature highlighting the importance of taking into account pretrial states and slower fluctuations. This can be critical especially for drug and neuroimaging studies as pharmacological agents may differently affect tonic and phasic noradrenaline and because different forms of baseline corrections in neuroimaging may have profound effects on the findings' interpretation.

At least one more study has used pupil size, as an indirect measure of NA, to study the role of fluctuations in NA in an uncertainty‐related decision process (Fan et al. 2023). Fan et al. found a positive correlation between pupil size and total uncertainty such that the choice stochasticity was associated with larger pupil size when the uncertainty was high. They did not observe a link between relative uncertainty and the pupil size. This might be due to their block design task containing task‐related information in the baseline pupil size, which would result in the baseline pupil size not being just purely endogenous fluctuations. Moreover, it needs to be highlighted that we characterised (un)certainty via the number of prior draws—in line with prior work (Dubois et al. 2021; Dubois and Hauser 2022; Wilson et al. 2014)—rather than the actual variance of the sample. This uncertainty reflects what is termed ‘ambiguity’ in the economics/risk taking literature, rather than ‘risk’, which would be an explicit probability of an outcome.

Although uncertainty is believed to govern different decision‐making strategies, our findings suggest that baseline pupil‐linked arousal is more likely to directly reflect uncertainty‐bonus related strategies (Auer 2003; Gershman 2018; Schwartenbeck et al. 2019), rather than stochasticity‐related mechanisms (Thomson 1933; Wilson et al. 2021), because we solely observe an effect on uncertainty and not on value. Latter mechanisms would imply that value would also be influenced by baseline pupil‐linked arousal, which we do not observe here. Moreover, the behaviour results showed that the choice likelihood over different value differences was shifted between the certain and uncertain choices, rather than being broadened, which is a typical characteristic of the stochastic‐related mechanism (Fan et al. 2023).

Here, we used (baseline) pupil size as a marking of NA functioning. Despite previous findings showing a link between pupillary responses and NA activity levels (Joshi et al. 2016; Joshi and Gold 2020), this association is only indirect, and it has been suggested that this link varies significantly across behavioural states (Allen et al. 2016; Bang et al. 2023; Megemont et al. 2022). Moreover, substantial evidence from animal and human studies suggests that phasic and tonic pupil size fluctuations covary not only with LC activity but also with activity of the basal forebrain, dopamine and raphe nuclei (Cazettes et al. 2021; de Gee et al. 2017; Hauser et al. 2019; Lloyd et al. 2023; Reimer et al. 2016). Thus, pupil size might also reflect activity of other neurotransmitters (e.g., serotonin, dopamine and acetylcholine). Further studies could use recent advances in recording more direct NA proxies in humans using methods such as fast cycling voltammetry (Bang et al. 2023) to further clarify the direct link between uncertainty processing and NA fluctuations.

Our findings show that subjects use uncertainty to guide their choices but that its impact is dependent on ongoing fluctuations in pupil size, a proxy for noradrenaline. Our finding that increased pupil‐linked arousal prior to trial onset renders humans more uncertainty‐avoidant means that the impact of uncertainty on decisions is enslaved by the continuous ebbs and flows of endogenous brain (noradrenaline) fluctuations.

## Author Contributions

**Ehsan Kakaei:** conceptualization, data acquisition, data curation, formal analysis, visualisation, writing of original draft. **Anne Schlecht:** data acquisition, data curation, formal analysis. **Tobias U. Hauser:** conceptualization, formal analysis, supervision, funding acquisition, investigation, reviewing and editing.

## Funding

This work was supported by Wellcome Trust (316955/Z/24/Z) and the H2020 European Research Council (946055).

## Conflicts of Interest

TUH consults for Limbic Ltd. and holds shares in the company, which is unrelated to the current project.

## Data Availability

The data that support the findings of this study are available under https://osf.io/pq8we. All analysis scripts are available under https://github.com/ehsankakaei91/Uncertainty_avoidance_pupillometry.
